# Flow situations during everyday practice in a medical hospital ward. Results from a study based on experience sampling method

**DOI:** 10.1186/1472-6955-10-3

**Published:** 2011-02-02

**Authors:** Åsa Bringsén, Göran Ejlertsson, Ingemar H Andersson

**Affiliations:** 1 School of Health and Society, Kristianstad University, Kristianstad, Sweden; 2 Department of Clinical Sciences Malmö, Faculty of Medicine, Lund University, Sweden

## Abstract

**Background:**

Nursing is a constant balance between strain and stimulation and work and health research with a positive reference point has been recommended. A health-promoting circumstance for subjective experience is flow, which is a psychological state, when individuals concurrently experience happiness, motivation and cognitive efficiency. Flow situations can be identified through individuals' estimates of perceived challenge and skills. There is, to the best of our knowledge, no published study of flow among health care staff. The aim of this study was to identify flow-situations and study work-related activities and individual factors associated with flow situations, during everyday practice at a medical emergency ward in Sweden, in order to increase the knowledge on salutogenic health-promoting factors.

**Methods:**

The respondents consisted of 17 assistant nurses and 14 registered nurses, who randomly and repeatedly answered a small questionnaire, through an experience sampling method, during everyday nursing practice. The study resulted in 497 observations. Flow situations were defined as an exact match between a high challenge and skill estimation and logistic regression models were used to study different variables association to flow situations.

**Results:**

The health care staff spent most of its working time in individual nursing care and administrative and communicative duties. The assistant nurses were more often occupied in individual nursing care, while the registered nurses were more involved in medical care and administrative and communicative duties. The study resulted in 11.5% observations of flow situations but the relative number of flow situations varied between none to 55% among the participants. Flow situations were positively related to medical care activities and individual cognitive resources. Taking a break was also positively associated with flow situations among the assistant nurses.

**Conclusions:**

The result showed opportunities for work-related interventions, with an adherent increase in flow situations, opportunity for experience of flow and work-related health among the nursing staff in general and among the assistant nurses in particular.

## Background

Nursing can be described as a constant balance between strain and stimulation [1] or stress versus satisfaction [2]. These descriptions can be related to what Csikszentmihalyi [3] calls the paradox of work. According to Csikszentmihalyi [3] work creates opportunities for powerful and enjoyable moments, but is nevertheless something that people often try to avoid. Many registered nurses have reported an intention to leave their nursing job [4,5] and studies of the hospital work environment pointed out problems in work design and management, with adherent work-related dissatisfaction, emotional exhaustion or strain [1,4].

It is, according to Kelloway, Teed and Kelley [6] as well as Theorell [7], time to shift the focus away from organisational shortcomings, illness and stress, towards organisational research with a more positive reference point. As work environments, health care settings seem to have a lot of positive characteristics, with adherent potential for important positive work-related experiences among health care staff [8-10]. Experiencing positive affects have been considered health-promoting in general [11] and temporary delights at work have been related to happiness, life satisfaction and psychological well-being [12].

Flow is considered the most advantageous circumstance for subjective experiences [13]. Flow has been described as a psychological state, when individuals concurrently experience happiness, motivation and cognitive efficiency [14]. The flow construct is multidimensional and there is an ongoing debate to whether flow is a state or traits construct [15]. The trait perspective emanates from the conclusion that individuals with an autotelic personality are more prone to the subjective experience of flow [16]. The state approach, on the other hand, means less stability and stronger relations to situational characteristics [15]. Previous research has shown that 74% of the variance in work-related flow was related to situational factors, when compared to dispositional features [15].

Flow situations can be measured through individuals' estimates of perceived challenge and skills in various everyday situations [17]. The initial research focused on chess players, rock climbers, rock dancers, and surgeons, based on the assumption that their challenging activities required highly skilled practitioners, with adherent opportunities for flow [13].

Studies have shown that flow among adults can be experienced more often in work situations than in leisure activities [12,18], but studies of flow in work settings are rare. Fullagar and Kelloway [15] demonstrated that flow mediated the relationship between work-related factors and employees' well-being and flow have also been related to employee performance [19].

To the best of our knowledge, there is no published study of flow among health care staff, despite their work being described as challenging and in need of highly skilled practitioners [20,21]. Previous research, focusing on workplace-related health resources in a nursing context, indicated that there ought to be opportunities for flow situations during health care workers everyday nursing practice [10].

The aim was to identify flow situations and study work-related activities and individual factors associated with flow situations, during everyday practice at a medical emergency ward, in order to increase the knowledge on salutogenic health-promoting factors.

## Methods

### Study Context and Participants

This study was carried out in a medium-sized Swedish hospital during September and October 2004. The study ward belonged to a care unit with 14 other wards and clinics. The ward specialised in gastroenterology and endocrinology and had a capacity to care for 24 patients. Health care staff at the ward consisted of 17 registered nurses, 21 assistant nurses and a receptionist (a trained assistant nurse), who took part in the health care work as well. A head nurse was in charge of the ward and two secretaries were managing various administrative duties. The caregivers were formally organized to work in pairs, consisting of one registered nurse and one assistant nurse during daytime shifts. Throughout the evening and night shifts, one registered nurse teamed up with two assistant nurses. Two teams were on duty during the evening shift and the ward was manned with one team throughout the night. Most of the staff had an individually controlled and flexible work schedule, so they could be scheduled for day shifts, evening shifts or night shifts. Fourteen registered nurses and 17 assistant nurses chose to participate in the study (n = 31, 79%) and six of them were only working night shifts.

### Procedure

The flow theory and the experience sampling method (ESM) [22], based on repeated measures, were used in the study. Since the aim was to study flow situations during everyday practice, we wanted to make sure that the different work-related activities representing "everyday practice" in this particular study context were covered. Prior to the study, the first author therefore made observations and took notes of the daily routines at the ward. The observations revealed a pattern of approximately two-hour periods or phases of work around the clock. These phases were characterised by the various routines that were in focus throughout different parts of an ordinary 24-hour period. The pattern was presented to and discussed with the staff at a staff meeting. They confirmed that the pattern was representative. The day and evening shifts were therefore divided into four periods of approximately two hours each, and the nightshift was divided into five periods. The participants got their own little booklet with several copies of a small questionnaire called an experience sampling form (ESF).

The participants completed one ESF, when they were randomly beeped through a preset wristwatch, once during each two-hour period. The time of signal was based on a random number table. The study period for each participant was one week and during this week, the participants could be scheduled to work three to six days. A limited number of wristwatches led to the division of the participants into four groups, doing the study consecutively. The total study period for the ward was therefore four weeks. The study resulted in a total of 497 ESFs, so the respondents completed the ESF an average 16 times (range 6 - 24 times). The internal drop out was 7% of the ESFs and most of the time a result of either sick leave, schedule changes or forgetting to put on the wristwatch during the study period.

At the beginning of each study period, the participants were also asked to complete the short version of Antonovsky's Sense of coherence (SOC) scale [23]. This was done in order to establish the respondents level of SOC, which is related to health and representative of individuals overall ability to manage stress [23]. Twenty five of the respondents filled in a SOC scale.

### Instruments

The ESF was first tested in a pilot study with fourteen members of the health care staff at the ward. The content was then discussed with the participants and the head nurse, as well as with a reference group consisting of researchers from the University and different representatives from the hospital. The final version of the ESF consisted of two questions on experienced challenges and skills, where the respondents were grading their experience on a ten-graded Likert scale (0-9). The ESF also had one question on time of signal and the possibility to report time for completing the ESF, if not doing this by the time of the signal. The ESF also contained one question on social context. Activity was covered by an open question, with the possibility for the respondents to describe what they were doing when they were signalled. So far the ESF was a revised Swedish version of a valid and reliable form presented by Csikszentmihalyi and Larson [22].

The ESF also consisted of nine questions that were focusing on the respondent's frame of mind and experience of cognitive performance indicators. These indicators had a preamble with an overall question: "To what extent do you feel..." followed by the respondents grading their experience of being concentrated, inventive, interested, dedicated, efficient, brisk, joyful, relaxed and pleased with their personal achievements. The rating of all the indicators was made on a ten-graded Likert scale (0-9). The frame of mind and experience of cognitive performance questions were developed for this study. These questions were later on further developed and included in a valid and reliable instrument for measuring salutogenic health indicators [24].

The respondents level of SOC was established using the short version of Antonovsky's SOC scale (13 items) [23] - the Swedish version. The SOC scale is considered a valid and reliable semantic differential, with questions covering the three comprehensive dimensions comprehensibility, manageability and meaningfulness [23].

### Variables

#### Flow situations

The participants' estimates of perceived challenge and skills formed the basis for a categorisation and identification of flow situations. In accordance with Ellis, Voelkl and Morris [25], flow situations were defined as challenge and skill estimation of five or higher on a ten-graded Likert scale (0-9), and an exact match between the two estimates. Flow situations were considered opportunities for flow experience. Due to the salutogenic focus of the study, the other combinations of answers were defined as non-flow situations with less opportunity for flow.

#### Time of day/Shift

The observed times of signal were divided between three categories of shift. Daytime was represented by signals from 06.45 to 15.30, evening consisted of signals from 15.31 to 22.00 and the night shift included signals from 22.01 to 06.44.

#### Social context

Social context was characterised by the respondents marking if they were alone or if someone else was present when the watch beeped.

#### Age

The dichotomisation of age was based on the median value of 39.

#### Profession

Profession was characterised by the two participating professional groups: assistant nurses and registered nurses.

#### Sense of Coherence (SOC)

SOC scores were studied through the short version of Antonovsky's SOC scale (13 items) [23] - the Swedish version. The scores for each question ranged from 1 to 7, thus the SOC value ranged from 13 to 91. The negatively directed scales were turned, so that the higher score on each question also meant higher value of SOC in total. The dichotomisation of SOC was based on the median value of 61.

#### Individual share of flow

Individual share of flow was calculated and dichotomised using the median level of 11%.

### Analyses

#### Qualitative categorisation of the activity descriptions

A qualitative categorisation of the participants' work-related activity descriptions was performed. The researcher (ÅB) went through the notes from the observations at the ward, and made a list of possible work-related activities with adherent descriptions and/or explanations. The list consisted of 25 different activities. Using this list, two external assistants took turns to identify different activities among the participants' answers. The researcher was available throughout the process, and if there were any uncertainties, the assistants discussed the classification with the researcher. The identified activities were then organised in categories representing different types of work-related activities, which were found to be representative for the everyday practice at the ward. The identified categories were then used in the forthcoming statistical analyses.

#### Statistical data preparation and analyses

The statistical part of the data preparation and analyses was done using the Statistical Package for Social Science (SPSS) 16.0 for Windows. A response level data file (n = 497) was used when working with response level data and an individual level data file (n = 31) made it possible to deal with the data in relation to the 31 participants. Individual level data and observational level data was analysed separately in order to avoid the problem with repeated measures counteracting the assumption of observations independence [17].

A Principal Component Analysis (PCA) was done, in order to explore the possibility of reducing the amount of variables but maintaining the content of the questions covering the participants' frame of mind and cognitive performance indicators throughout the analysis. The varimax method for orthogonal rotation was used for factor extraction and Eigenvalue > 1 was set as a criterion. Two indexes were constructed emanating from the result of the PCA, and these indexes were then used throughout the statistical analysis. The internal consistency of the indexes was studied using Cronbach's Alpha (CA). The categorisation of the index values into high and low was based on the median value.

Differences between groups were tested with chi-squared test (categorical variables), and Mann-Whitney U-test (numerical variables). A logistic regression model was used to study different variables' relationship to flow. Variables with p < 0.25, when bivariately related to flow, were included in the analysis. Results were presented as odds ratios (OR) with 95% confidence intervals. Logistic regression models were performed on the observational data as a whole and on the observational data from the assistant nurses. The observational data from the registered nurses was too small for a logistic regression analysis. The significance level was set to 0.05 for all tests.

The definitions of variables in the logistic regression analysis are presented in Table 1.

**Table 1:** Variables in the logistic regression analysis of observational data, with flow situation as the dependent variable.

Variable	**Type of data **^**a)**^	Categorisation
Activity	N	Individual nursing care, Medical care, Administration and communication, Taking a break, Other activities
Cognitive resources (Index)	Num	High (> 34), Low (34 and below)
Flow situation	N	Flow situation, Non-flow situation

^a) ^N = Nominal, Num = Numerical

### Ethical Issues

The study was carried out in accordance with Swedish law of research ethics (SFS 2003:460) and the study was approved by the Research Ethics Committee of Lund University (Dnr: 279/2004).

## Results

The mean age of the 31 participants was 40. Assistant nurses had a mean age of 48, and the registered nurses' mean age was 31. A total of 497 ESFs, or observations, was collected throughout the study period, and assistant nurses completed 267 (53.7%) of these, wile the registered nurses completed the other 230 (46.3%). The PCA resulted in a two-factor model dividing the cognitive- and affect-related variables into two categories. One category included the five variables concentrated, inventive, interested, dedicated and efficient, named *cognitive resources*. The other category contained the remaining four variables brisk, joyful, relaxed and pleased with personal achievement, called *affective resources*. The result from the PCA and the reliability analysis of the categories are presented in Table 2.

**Table 2:** Results from the principal component analysis of frame of mind and cognitive ability variables.

Variable	Cognitive resources (CR)	Affective resources (AR)
	Factor loadings	Factor loadings
Concentrated	**0.85**	0.12
Inventive	**0.70**	-0.01
Interested	**0.76**	0.32
Dedicated	**0.83**	0.32
Efficient	**0.72**	0.30
Brisk	0.34	**0.77**
Joyful	0.29	**0.81**
Relaxed	-0.07	**0.87**
Pleased with personal achievement	0.24	**0.67**

Bold figures indicate factor belonging.

Communalities varied between 0.49-0.80. Eigenvalue CR = 4.44, AR = 1.57. Determinant = 0.009, Kaiser-Mayor-Olkin measure of sampling adequacy = 0.84, Bartletts's test of sphericity = 0.00 and explained variance = 66.8%. Cronbach's alpha CR = 0.86, AR = 0.82

Twenty two work-related activities were identified in the ESFs, and these were organised in five categories representing different types of work-related activities:

1. *Individual nursing care *was characterised by tasks where the health care staff was caring for the patients in a general sense, and when they were involved in other surrounding routines supporting the caring of the patients in general.

2. *Medical care *was based on activities that were linked to the specific medical care of the patients.

3. *Administration and communication *consisted of different sorts of paper work, as well as planning and communication activities in general.

4. *Taking a break *consisted of observations when the staff was at work but not working.

5. *Other activities *were situations when the staff was in-between the other categories of work-related tasks or involved in helping students at the ward.

**Figure 1:**
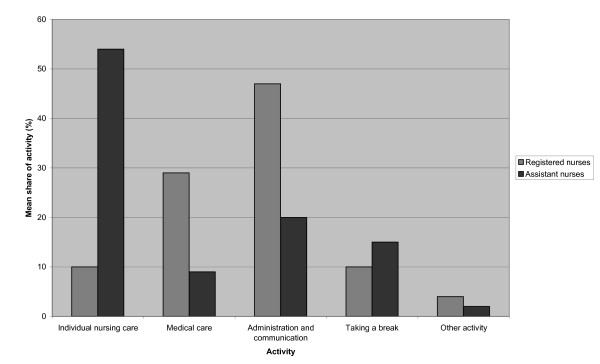
**Mean share of activities for registered nurses and assistant nurses (%)**.

The health care staff spent most of its working time in individual nursing care, followed by performing administrative and communicative duties. There were significant differences when comparing the two professional group's mean percentage of being involved in different activities (n = 31). The assistant nurses were more often occupied in individual nursing care (p = 0.000), while the registered nurses were more frequently involved in medical care (p = 0.000), as well as performing administrative and communicative duties (p = 0.000). The distribution of activities in the two groups is shown in Figure 1.

Flow situations were registered during 57 (11.5%) observations, and at an individual level, flow situations were represented in between none and 55% of the participants' total number of observations.

**Figure 2:**
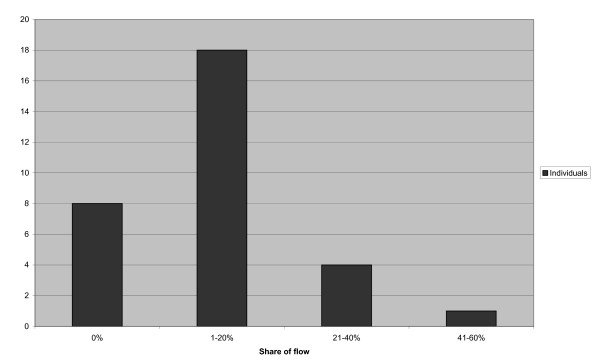
**Number of individuals with different proportions of flow situations**.

As can be seen in Figure 2, some of the participants did not have any observed flow situation while others had a higher proportion of registered flow situations. There was no significant association between individuals' share of flow situations and variables such as age group (p = 0.873), profession (p = 0.444) or SOC level (p = 0.107).

The initial bivariate analysis of different variables relationship to flow situations (n = 497), revealed significant associations with activity (p = 0.023) and cognitive resources (p = 0.010). A presentation of the variables included, and the total results from the analyses, are presented in Tables 3 and 4.

**Table 3:** Flow situations in relation to different shifts, activities and social contexts.

Variable	Categories (number of observations)	Observations of flow situations/non-flow situations	% of flow	**p value**^**a**^
Shift	Daytime (n = 266)	30/236	11.3	
	Evening (n = 121)	15/106	12.4	
	Nightshift (n = 109)	11/98	10.1	0.859
Social context	Alone (n = 262)	32/230	13.9	
	Someone else present (n = 235)	25/210	10.6	0.582
Activity	Nursing (n = 169)	12/157	7.1	
	Medical care (n = 92)	19/73	20.7	
	Administration and communication (n = 162)	18/144	11.1	
	Taking a break (n = 55)	6/49	10.9	
	Other activities (n = 16)	1/15	6.3	**0.023**

^a ^Results from chi square test. Bold when significant.

**Table 4:** Mean (SD) for cognitive and affective resources in flow situations and non-flow situations.

Variable (n)	Flow (SD)	Non-flow (SD)	p value
Cognitive resources (index n = 493)	35.0 (7.32)	31.7 (9.01)	**0.010**
Affective resources (index n = 497)	28.1 (5.85)	28.7 (5.75)	0.394

Bold figures indicate significant values.

The results from the logistic regression analyses are presented in table 5. The findings showed that being involved in medical care had the strongest relationship to flow situations, with an OR of 3.21. Reporting high on cognitive resources was also positively related to flow situations (OR = 1.80).

**Table 5:** Results from logistic regression analyses with flow situation as the dependent variable.

Variable	Category	All participants OR (CI)	Assistant nurses OR (CI)
Activity	Individual nursing care	1.0	1,0
	Medical care	**3.21 (1.47-7.00)**	**3.25 (1.06-9.90)**
	Administration and communication	1.61 (0.75-3.46)	0.82 (0.25-2.69)
	Taking a break	1.84 (0.65-5.24)	**3.06 (1.01-9.27)**
	Other activities	0.85 (0.10-7.03)	^a^--------
Cognitive resources (index)		**1.80 (1.00-3.23)**	**2.44 (1.02-5.81)**

Bold figures indicate significant values. Variables with p > 0,25, when related to flow, were not included in the analysis. The excluded variables were time of day/shift, social context and affective resources.

Model fits:

All participants: Nagelkerke = 0.057, Hosmer and Lemeshow test p = 0.968, Prediction = 88.7%

Assistant nurses: Nagelkerke = 0.096, Hosmer and Lemeshow test p = 0.950, Prediction = 89.5%

^a ^There were too few observations in this activity category.

There was also a positive relationship between flow situations and medical care, as well as cognitive resources, when focusing on assistant nurses only (OR = 3.25 and 2.44, respectively). In this analysis, taking a break also had a positive relationship to flow situations, with an OR of 3.06.

## Discussion

The empirical application of flow situations used in this study resulted in 23 participants being in a flow situation on different occasions, and 11.5% of the total number of observations was flow situations. Flow at work can be linked to work-related employee enjoyment, since individuals who enjoy their work are more often reporting flow at work [12,16,18,26]. However, studies on the hospital work environment have instead been highlighting problems in work design and management, with adherent work-related dissatisfaction, emotional exhaustion or strain [1,4]. From our point of view, it is likely that these problems limit the possibility for work-related enjoyment among nursing staff in general, indicating a possibility for improvement of the work environment with adherent facilitation of flow situations. We found no significant relationship between flow situations and social context in our study, a result that is supported by Allison's and Duncan's [27] conclusion that flow can be experienced alone, as well as in interaction with others.

Csikszentmihalyi [13] talked about autotelic personality individuals being more likely to experience flow, and Eisenberger et al. [19] showed that flow, among achievement-oriented employees only, was associated with a positive mood as well as task interest and performance. These personality traits may explain the individual differences in observed flow situations within our material. We could not, however, find any relationship between share of observed flow situations and age group, profession or SOC level. A variation in flow-related personality traits is thus probably not related to any of the individual variables used in our study. The lack of a relationship between share of flow situations and individual characteristics can also be a result of the small material on individual level in the study.

Flow situations has been more often observed at work than during leisure [18,26]. Allison and Duncan [27] found that work, as well as non-work situations, was a resource for flow among professional female workers and that flow among blue collar workers was linked to non-work situations to a greater extent [27]. There was no direct relationship between profession and individual share of flow situations in our study. However, the relationship between taking a break and flow situations, when focusing on the assistant nurses only, is considered supportive of the idea of professionally related differences in flow opportunities among female workers. A previous study described the character of taking a break, as well as the relationship between the assistant nurses, in a way that is considered similar to social (non-work) situations in general [10].

Another indirect link between profession and flow opportunities can be related to the activity-associated results in this study. The results showed that being involved in medical care was positively related to flow situations, and the registered nurses reported being occupied in medical care more often than the assistant nurses. The profession-related activity difference indicates that registered nurses generally have better opportunities for flow experiences, since registered nurses in Swedish health care organisations are normally more occupied in medical care [21]. Blue-collar workers generally have less opportunity for flow when they are at work according to Allison and Duncan [27]. Profession-related differences can be a result of individual characteristics, if for instance individuals with an autotelic personality are more prone to becoming registered nurses or professionals in general. It is thus more likely that the profession-related differences are results of work-related situational factors, since 74% of the variance in flow has been linked to situational characteristics when compared to dispositional features [15].

We found a relationship between cognitive resources and flow situations which can be considered a validation of the cognitive and partly motivational components of the flow construct. However, we found no significant association between affective resources and flow situations. This pattern can be recognised from the work of LeFevre [28], who also found that people in general experience more positive affect the more time they spent in a state of flow. This means that if the opportunities for flow were increasing and the participants in our study were to experience flow in the flow situations, they would also experience positive affects more frequently, with adherent possibilities for health. Different studies have reported a relationship between positive mood and flow [15,19] and flow seems to be important for the psychological well-being in general [16]. Experiencing positive affects have also been considered health-promoting in a general sense [11].

The empirical findings in this study indicate that research focusing on flow can be an important contribution to the research field of work and health in nursing contexts. Focusing on the positive side of nursing practice, like for instance flow, means knowledge development of the salutogenic components of nursing. These components give opportunity for a nursing practice characterised by positive aspects instead of the traditional problem-oriented and pathogenic focus. Nursing practice with a positive frame of reference is a way of promoting the health of health care workers and improves the opportunity for high quality nursing practice [10]. This will hopefully make nursing practice more attractive and reduce the amount of registered nurses who want to leave their nursing jobs. Further research is needed for an empirical assessment and exploration of relationships between health, subjective experiences of flow in general and affective resources in particular and flow situations in nursing, as they have been operationalized in our study.

Using the ESM in a hospital setting is a complicated and challenging endeavour. The design of the study resulted in a mix of quantitative and qualitative planning and preparation phases. The mix of qualitative and quantitative phases throughout the research process fits Johnson and Onwuegbuzie's [29] description of a mixed model approach in mixed method research. The initial observations at the ward facilitated the validity because of the adherent possibility to make sure that the participants' everyday practice was systematically covered in the study. The results from the observations were also useful and increased the validity in relationship to the labelling and categorising of the activity responses. The qualitative steps, with adherent researcher-subjective interpretation, could, however, be considered a limitation of the study's reliability. The participants' verification of the observation findings has hopefully, on the other hand, limited any possible negative impact on the reliability. ESM with an ESF is considered a valid and reliable method for measuring various dimensions of experience [22] and useful for studying everyday life in general [17]. However, the research process needs a number of different steps in order to yield a manageable data material with the possibility for valid and reliable results in the end. We have tried to describe the process thoroughly, to help the readers understand what has been done and judge the credibility of the findings.

Most of the respondents have previously reported having a normal everyday life throughout an ESM study period [22], and in our study, participation was found to be manageable and somewhat amusing during everyday work. The within context character of the study reduces the risk for recall bias and the ecological validity is strengthened by a habituation process, related to respondents completing the Experience Sampling Form (ESF) repeatedly [17]. One of the problems with repeated measures is that it counteracts the assumption of observations independence [17]. In this study, the problem was solved by differentiating between individual level data and response level data, and analysing them separately. Using individual level data is recommended [17], but the small number of participants in this study (n = 31) limited the possibility for advanced analysis of the individual level data.

Measurement of flow situations through people's estimates of challenges and skills can be, and have been, done in numerous and varying ways [17], resulting in inter-study comparison limitations. In this study, we have chosen to use raw scores and defined the flow situation as estimates above five on the ten-graded Likert scale (0-9) and a perfect match between the respondents' answers on challenge and skills. This definition of the flow situation is recommended, since it acknowledges individual differences in the data and is considered to be in line with the respondents' opinion on what constitutes a match between challenge and skill estimation [25]. From our point of view, flow situations should be a relatively rare phenomenon, since flow is described as a subjective experience of enjoyment, complete absorption and a moment where capabilities are being stretched to facilitate learning, and increase self esteem and personal complexity [3,18].

In most studies of flow, items for constructing affect and arousal indexes are included [25]. In this study, the items included were categorised as cognitive and affective resources, respectively. Customary use of indexes of affect and arousal in studies of flow, despite rather low values of internal consistency, has been criticised [25]. The indexes used in this study were, however, based on a PCA with good quality according to the psychometric properties, and the Cronbach's alpha values were as high as 0.86 and 0.82, respectively.

## Conclusions

This first study of flow opportunities among health care staff identified positive relationships between flow situations and medical care activities, cognitive resources as well as taking a break. More research is needed in order to further explore the nature of these identified relationships. However, the results indicate a potential for work-related intervention in order to increase the opportunity for flow situations, experience of flow and work-related health among health care staff in general, and among the assistant nurses in particular. Refocusing the health care work towards more medical care activities and less administration, for instance, could result in more flow opportunities, with adherent possibilities for improved health among the staff. The findings, together with further research, facilitate development of a nursing practice from a positive frame of reference. This gives opportunities for a promotion of the health of health care workers and facilitates high quality nursing practice. Nursing practice can thereby become more attractive and the number of registered nurses who want to leave their nursing jobs can be reduced.

## Competing interests

The authors declare that they have no competing interests.

## Authors' contributions

ÅB was main responsible for the planning and implementing of the study as well as the analysis and the drafting of the manuscript. IHA functioned as a support in the planning of the study, participated actively in the statistical analysis and helped in drafting the manuscript. GE functioned as a support in the planning, throughout the statistical analysis and helped in drafting the manuscript. All authors read and approved the final manuscript.

## Pre-publication history

The pre-publication history for this paper can be accessed here:

http://www.biomedcentral.com/1472-6955/10/3/prepub
